# Mumps Seroprevalence in Vellore, South India: A Community-Based Cross-Sectional Study

**DOI:** 10.4269/ajtmh.25-0069

**Published:** 2025-08-12

**Authors:** Ramya Madhavan, Tintu Varghese, Akilandeswari Eswaran, Vaishnavi Gandhi, Poornima Saravanan, Lakshmi Raj, Reshma Raju, Julian Vivek Leander Xavier, Jovin Stanley Joseph, Prasanna Samuel Premkumar, Winsley Rose, Jacob John

**Affiliations:** ^1^ The Wellcome Trust Research Laboratory, Christian Medical College, Vellore, India;; ^2^ Department of Child Health, Christian Medical College, Vellore, India;; ^3^ Department of Biostatistics, Christian Medical College, Vellore, India;; ^4^ Department of Community Medicine, Christian Medical College, Vellore, India

## Abstract

The mumps vaccine remains excluded from India’s National Immunization Schedule (NIS) because of insufficient data on the community-level disease burden, despite mumps being a vaccine-preventable disease. In the present study, biobanked serum samples from an urban population in Vellore, stratified by age (in age groups of 1–5, 6–15, and 16–40 years) and sex, were tested for anti-mumps IgG antibodies using ELISA. The seroprevalence rates of mumps were 26%, 71%, and 84% in the 1- to 5-year, 6- to 15-year, and 16- to 40-year age groups, respectively, indicating a high burden in the community. Women in the 16- to 40-year age group exhibited a higher seroprevalence compared with males, with antibody levels consistently lower in males across both the 6- to 15-year and 16- to 40-year age groups (*P* <0.05). The present study highlights increasing mumps exposure with age and underscores the urgent need to include the mumps vaccine in the NIS to address the high disease burden and prevent complications.

## INTRODUCTION

Mumps is a contagious viral infection caused by the mumps virus and clinically characterized by fever and swelling of the salivary glands. Well-documented complications, such as aseptic meningitis (15%), encephalitis (1%), sensorineural hearing loss (4%), orchitis (30–40%) in pubertal and post-pubertal males, and abortion in females, contribute to the disease’s public health impact.^1^^,^^2^ Since the introduction of the first mumps vaccine (Jeryl Lynn strain) in 1967, 121 countries have integrated it into their routine immunization programs.^3^ While sporadic outbreaks still occur in vaccinated populations, the severity of complications is markedly reduced compared with unvaccinated individuals.^4^ There have been recurring mumps outbreaks in many regions of India, with the most recent ones being reported in Kerala, Tamil Nadu, Karnataka, Odisha, Chhattisgarh, and Navi Mumbai in 2023.^2^^,^^5^^,^^6^ These outbreaks have predominantly affected school-aged children and young adults, highlighting the urgent need for vaccination in these target populations.

The Indian National Immunization Schedule (NIS) includes measles and rubella vaccine but excludes the mumps vaccine. The IDsurv system, managed by the Indian Academy of Pediatrics (IAP), reveals sporadic mumps outbreaks as reported by pediatricians, whereas the Integrated Disease Surveillance Program (IDSP) collects weekly mumps data from primary health centers and health subcenters. However, these reported cases likely underrepresent the true burden because many subclinical infections go unnoticed and unreported. In a study conducted in Kashmir as part of the IDSP, mumps outbreak data were analyzed, and 15 outbreaks were identified over a 3-month period. The study highlighted the need to strengthen mumps surveillance to support global health security.^7^ In addition to inadequate surveillance, the limited number of studies on comprehensive community-level data regarding mumps prevalence remains a critical barrier to include the vaccine in India’s NIS.^3^ In this context, we conducted a mumps seroprevalence study to evaluate the disease burden in Vellore, South India, with an age- and sex-stratified analysis. The findings are aimed at identifying at-risk groups and contributing evidence to inform decision-making on mumps vaccination in India.

## MATERIALS AND METHODS

This study used bio-banked serum samples collected from a cross-sectional study to estimate typhoid seroincidence in the community in 2022 in Vellore, South India. Blood samples were collected from individuals aged up to 40 years residing in the five urban clusters within the Vellore Demographic Health Surveillance System area. Participants included individuals residing in the study area who would remain there for a duration of the 2-year study and who provided informed consent. Participants with a history of prolonged steroid use or other immunodeficiency were excluded at the time of the serosurvey. Vaccination status was only available for children 1–5 years of age. We estimated a sample size of 700 based on a seroprevalence of 75.5% among children aged 5–10 years, with a 95% CI and a precision of 5%.^8^ The sampling was conducted by using a stratified random sampling method, in which every fourth sample on the list was selected within each age stratum. Of a total of 5,000 samples, 723 were chosen using this method. The participants were subdivided into three age groups: 1–5 years, 6–15 years, and 16–40 years of age. A total of 143 samples from individuals aged 1–5 years (82 females and 61 males), 295 samples from the 6- to 15-year age group (146 females and 149 males), and 285 samples from individuals aged 16–40 years (145 females and 140 males) were tested using an anti-mumps IgG enzyme immunoassay (Euroimmun AG, Lubeck, Germany, EI 2630–9601 G).^9^ Mumps IgG serostatus was defined according to the manufacturer’s guidelines: ≥22 relative units (RU)/mL indicates seropositivity, <16 RU/mL indicates seronegativity, and 16 to <22 RU/mL indicates an equivocal result. Antibody levels were measured by plotting extinction readings from three calibration sera point by point. The assay has a reported sensitivity of 99.6% and specificity of 100%.^9^ Equivocal samples were reanalyzed in duplicate using the same assay. The final result for each sample was determined on the basis of the most frequently occurring outcome among the three qualitative results. Equivocal samples were considered positive in the primary analysis. In addition, a sensitivity analysis was performed by treating equivocal results as negative. Age, sex, and cluster-specific associations with serostatus were analyzed by using the χ^2^ test, whereas antibody levels were compared across age groups by using the Kruskal–Wallis test and between sexes with the Mann–Whitney *U* test. All statistical analyses were performed by using R (v4.4.1, R Foundation for Statistical Computing, Vienna, Austria).

## RESULTS

**Figure 1.**
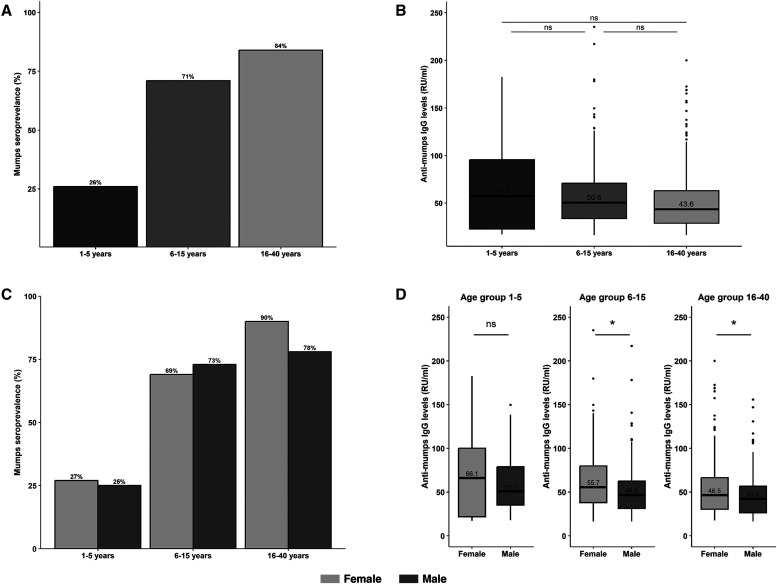
Mumps seroprevalence and anti-mumps IgG antibody levels stratified by age and sex in an urban population from Vellore, India. (**A**) Overall seroprevalence of mumps across the three age groups (1–5 years, 6–15 years, and 16–40 years). (**B**) Distribution of anti-mumps IgG antibody levels (relative units [RU]/mL) across these age groups. (**C**) Comparison of mumps seroprevalence between males and females within each age group. (**D**) Sex-based distribution of anti-mumps IgG antibody levels (RU/mL) for the three age groups, with statistical significance indicated (*P <0.05; ns = not significant). Bars and boxplots represent proportions and median antibody levels, respectively, along with interquartile ranges and outliers. Antibody levels were compared across age groups by using the Kruskal–Wallis test and between sexes with the Mann–Whitney *U* test.

Among 143 children aged 1–5 years, 50 had received two doses of the measles vaccine (measles-containing vaccine), 79 had received two doses of the measles and rubella (MR) vaccine, and three children had not been vaccinated. None of them had received the measles, mumps, rubella (MMR) +/– varicella (V) vaccine. Among this age group, seropositivity was lowest at 26% (95% CI: 19–33), indicating a susceptibility of 74% to mumps. In contrast, the 6- to 15-year age group exhibited a seropositivity of 71% (95% CI: 66–76), and the highest seropositivity was observed in the 16- to 40-year age group at 84% (95% CI: 80–88; Figure 1A and Table 1). When the data were stratified by sex, seropositivity did not differ significantly between males and females in either of the lower age groups. In the 1- to 5-year age group, the seropositivity rates were 27% (95% CI: 17–37) in females and 25% (95% CI: 14–36) in males (*P* = not significant [ns]). In the 6- to 15-year-age group, the rates were 69% (95% CI: 61–77) in females and 73% (95% CI: 66–80) in males (*P* = ns). However, in the 16- to 40-year age group, seropositivity was significantly higher in females (90% [95% CI: 85–95]) compared with males (78% [95% CI: 71–85]; *P* = 0.01; Figure 1C and Table 1). The sensitivity analysis revealed a similar trend when equivocal results were treated as negative (Supplemental Figure 1). The results of repeat testing for equivocal samples, along with the corresponding seroprevalence estimates of mumps when these samples are considered either positive or negative, are presented in Supplemental Tables S1 and S2, respectively.

**Table 1:** Seroprevalence of mumps IgG antibodies stratified by age group, sex, and geographic cluster in an urban population from Vellore, India

Age Group (years)	Demographics	Seropositive, *N* (%)	Seronegative, *N* (%)	*P*-Value
	1–5	37 (26)	106 (74)	<0.05
	6–15	210 (71)	85 (29)	
	16–40	240 (84)	45 (16)	
1–5	Female	22 (27)	60 (73)	0.91
	Male	15 (25)	46 (75)	
6–15	Female	101 (69)	45 (31)	0.53
	Male	109 (73)	40 (27)	
16–40	Female	131 (90)	14 (10)	<0.05
	Male	109 (78)	31 (22)	
1–5	Cluster 1	8 (22)	29 (78)	0.66
	Cluster 2	5 (19)	21 (81)	
	Cluster 3	8 (25)	24 (75)	
	Cluster 4	10 (34)	19 (66)	
	Cluster 5	6 (32)	13 (68)	
6–15	Cluster 1	47 (76)	15 (24)	0.10
	Cluster 2	49 (75)	16 (25)	
	Cluster 3	39 (71)	16 (29)	
	Cluster 4	31 (56)	24 (44)	
	Cluster 5	44 (76)	14 (24)	
16–40	Cluster 1	49 (78)	14 (22)	0.22
	Cluster 2	52 (90)	6 (10)	
	Cluster 3	47 (80)	12 (20)	
	Cluster 4	44 (85)	8 (15)	
	Cluster 5	48 (91)	5 (9)	

Data are presented as the number (*N*) and percentage (%) of seropositive and seronegative individuals within each subgroup. *P*-values indicate the statistical significance of differences observed within the respective categories.

**Figure 2.**
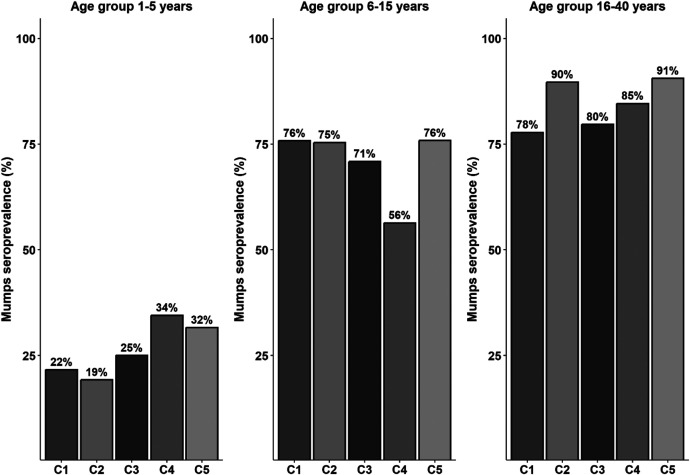
Mumps seroprevalence stratified by age group (1–5 years, 6–15 years, and 16–40 years) and geographical cluster (C1–C5) in an urban population from Vellore, India. Bars indicate the percentage of seropositive individuals in each cluster (C1 = Cluster 1: Kaspa; C2 = Cluster 2: Vasanthapuram; C3 = Cluster 3: Shenpakkam; C4 = Cluster 4: Mullipalayam; C5 = Cluster 5: SSk Maniyam).

IgG antibody levels, when analyzed across sex and age groups, exhibited additional patterns. In the 1- to 5-year age group, IgG antibody levels (measured in RU/mL) did not differ significantly between females and males. The median IgG levels were comparable (the median [interquartile range (IQR)] was 66.1 [21.8–100.1] in females versus 50.7 [35.1–78.9] in males; *P* = ns). In the 6- to 15-year age group, IgG levels were significantly higher in females (median [IQR] 55.7 [37.9–79.9]) than in males (median [IQR] 46.6 [31.3–62.5]; *P* <0.05). A similar trend was observed in the 16- to 40-year age group, where females exhibited significantly higher median IgG levels (median [IQR] 46.5 [30.3–66.6]) compared with males (median [IQR] 42.4 [26.3–56.8]; *P* <0.05; Figure 1D). There was no difference in mumps seroprevalence between the urban clusters because they were homogeneous (Table 1 and Figure 2).

## DISCUSSION

Mumps has emerged as a significant public health concern because of numerous outbreaks reported globally, including in India, in recent years. The NIS in India does not include the mumps vaccine, despite the recommendations by the IAP regarding the use of the MMR or MMRV vaccine in a two-dose schedule at 9–12 months and 16–24 months of age.^3^

The present study reveals that mumps seroprevalence increases with age, with the highest rate observed in the 16- to 40-year age group (84%). The lowest seroprevalence rate was found in children in the *≤*5-year age group (26%), indicating that this age group is highly vulnerable to mumps, particularly when they begin kindergarten. Studies from Sudan, Iran, and Tanzania have revealed much higher seroprevalence rates among unvaccinated children under 5 years of age, ranging from 63% to 80%.^10^^–^^12^ However, the seroprevalence may have been overestimated in these hospital-based studies because of sampling bias. Our findings indicate an ongoing circulation of the virus in the community, with an increasing incidence of infection with age. We also investigated whether past mumps outbreaks or vaccination campaigns might explain the differences in seroprevalence rates among the age groups. A review of the IDSP and other outbreak reporting databases revealed no documented mumps outbreaks or targeted vaccination campaigns in the Vellore District. Therefore, the observed age-related differences in seroprevalence are unlikely to be influenced by previous outbreaks or vaccination efforts specific to the region.

Although antibody levels did not vary significantly with age, males consistently exhibited significantly lower antibody levels compared with females (*P* <0.05). Previous studies among vaccinated individuals have similarly revealed higher neutralizing antibody levels in females than males,^13^^,^^14^ which have been attributed to sex-linked genetic differences, including variations in human leukocyte antigen and cytokine receptor gene polymorphisms.^14^ While direct evidence in unvaccinated individuals is limited, sex-based differences in immune responses have been observed across various infections,^15^ suggesting that similar patterns may exist. However, further research is needed to determine whether these lower antibody levels in males influence susceptibility to mumps-related inflammation or disease severity.

Testicular inflammation caused by mumps can disrupt the blood–testis barrier, leading to the production of autoantibodies against immune-privileged antigens. While sterility from mumps orchitis is rare, subfertility is more common, with oligospermia or azoospermia reported.^16^ Fertility impairment occurs in 13% of unilateral orchitis cases and 30–87% of bilateral orchitis cases.^16^^,^^17^ However, data from India are limited because of inadequate mumps surveillance. The complications and health impacts associated with mumps are a major public health concern.

Although the mumps vaccine is available in the private sector as the MMR/MMRV vaccine, 94.5% of children in India receive vaccines through public health facilities, which do not include the mumps vaccine.^18^ Studies from countries like the United States^19^ and Japan^20^ have highlighted the cost-effectiveness of national mumps vaccination, reducing school and workplace absenteeism, as well as complications. Given the high community-level seroprevalence in the present study, the findings support the IAP’s recommendation to include the mumps vaccine in the NIS alongside the MR vaccine. Although cost-effectiveness data for India regarding the mumps vaccine are lacking, a study conducted in Odisha, India, revealed that vaccinating the community with the mumps vaccine could have reduced costs by 35% compared with outbreak-related expenses.^2^

The main limitation of the present study is the absence of vaccination information for individuals older than 5 years and clinical data for all participants. However, because most vaccinations in India are administered through government programs and none of the 1- to 5-year age group in this urban population received the MMR vaccine, it is assumed that the seroprevalence rates in other age groups reflect the true disease burden of mumps. In addition, the samples were collected for a typhoid seroincidence study and were not intended for measuring mumps seroprevalence, which limits the generalizability of the findings.

## CONCLUSION

This is the first community-based study in India to stratify mumps seroprevalence by age and sex and highlights increasing mumps exposure with age. Future multicenter studies should be conducted to assess the national burden to inform the inclusion of the mumps vaccine in India’s immunization schedule.

## Supplemental Materials

10.4269/ajtmh.25-0069 Supplemental Materials
