# Supplementary material for: Mumps Seroprevalence in Vellore, South India: A Community-Based Cross-Sectional Study
Source: Am J Trop Med Hyg. 2025 Aug 12;113(4):883–7. doi: 10.4269/ajtmh.25-0069 (PMC12493236; doi:10.4269/ajtmh.25-0069)
Supplement: Supplemental Materials [file tpmd250069.SD1.pdf]

Supplemental Figure S1: Sensitivity analysis considering equivocal results as negative. (A). Overall mumps seroprevalence across three age groups (1–5-, 6–15-, and 16–40-year age group) (B). Comparison of mumps seroprevalence between males and females within each age group.

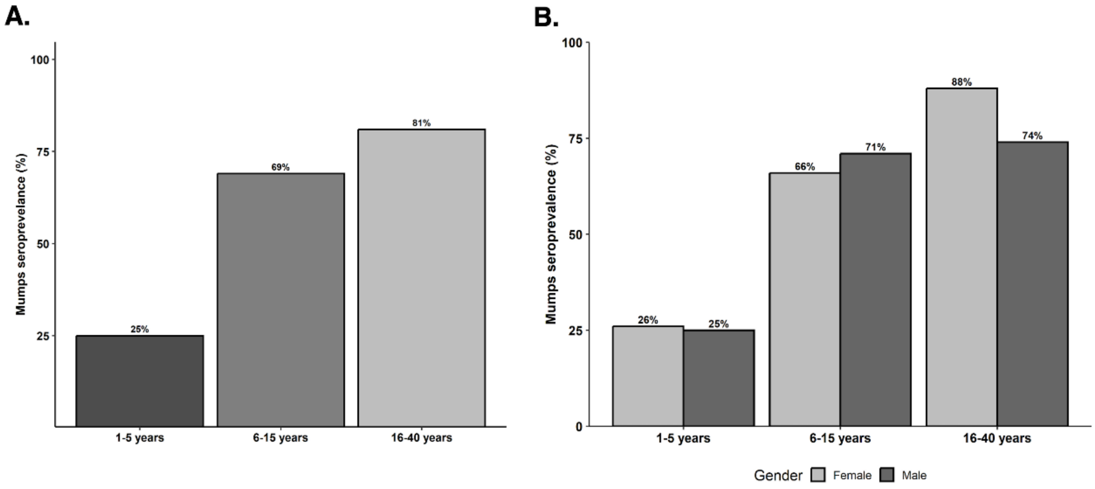

Figure S1: *Sensitivity analysis considering equivocal results as negative.*
(A). Overall mumps seroprevalence across three age groups (1–5, 6–15, and 16–40 years)
(B). Comparison of mumps seroprevalence between males and females within each age group

Supplemental Table S1

Reports of equivocal results and duplicate retest outcomes of equivocal samples

Test result	*N* (%)
Number of Equivocal test result (first time)	39
Equivocal retest results as Equivocal (done in duplicates)	17 (50%)
Equivocal retest results as positive (done in duplicates)	15 (38%)
Equivocal retest results as negative (done in duplicates)	7 (17%)

Supplemental Table S2

Seropositivity percentages for the primary outcome and sensitivity analysis, stratified by age and gender. The table presents the proportion of individuals seropositive for the primary outcome measure, along with results from sensitivity analyses

**Age Strata**	**Seropositivity (equivocal as seropositive in the primary outcome)** ***N* (%)**	**Seropositivity (equivocal as seronegative in the sensitivity analysis)** ***N* (%)**
1–5	37 (26%)	36 (25%)
6–15	210 (71%)	203 (69%)
16–40	240 (84%)	231 (81%)

**Gender Strata**	**Seropositivity (equivocal as seropositive in the primary outcome)** ***N* (%)**	**Seropositivity (equivocal as seronegative in the sensitivity analysis)** ***N* (%)**
1–5 (male)	15 (25%)	15 (25%)
1–5 (female)	22 (27%)	21 (26%)
6–15 (male)	109 (73%)	106 (71%)
6–15 (female)	101 (69%)	97 (66%)
16–40 (male)	109 (78%)	103 (74%)
16–40 (female)	131 (90%)	128 (88%)
